# A prediction model for post-treatment presence of coronary artery abnormality before initial treatment in Kawasaki disease in Japan

**DOI:** 10.3389/fped.2025.1647195

**Published:** 2025-12-02

**Authors:** Takayuki Suzuki, Naomi Kitano, Nobuyuki Kakimoto, Tomohiro Suenaga, Shoichi Shibuta, Takashi Takeuchi, Hiroyuki Suzuki, Kota Abe, Kumi Yasukawa, Kentaro Okunushi, Hiromichi Hamada, Daisuke Tokuhara

**Affiliations:** 1 Department of Pediatrics, School of Medicine, Wakayama Medical University, Wakayama, Japan; 2 Division of Public Health and Health Management Center, School of Medicine, Wakayama Medical University, Wakayama, Japan; 3 Division of Pediatrics, Kinan Hospital, Tanabe, Japan; 4 Division of Pediatrics, Kainan Municipal Hospital, Kainan, Japan; 5 Division of Pediatrics, Tsukushi Medical and Welfare Center, Iwade, Japan; 6 Division of Pediatrics, Tokyo Women’s Medical University Yachiyo Medical Center, Yachiyo, Japan; 7 Department of Pediatrics, Graduate School of Medicine, Chiba University, Chiba, Japan

**Keywords:** coronary artery abnormality, coronary artery *z*-score, echocardiography, Kawasaki disease, predictors

## Abstract

**Background:**

This study aimed to develop and validate a predictive model for the presence of coronary artery abnormality (CAA) after treatment using pre-treatment clinical and laboratory parameters, including coronary *Z*-scores, in Japanese patients with Kawasaki disease (KD).

**Method and results:**

A retrospective multicenter cohort study was conducted, analyzing 1,565 patients diagnosed with KD across eight medical institutions within the Wakayama Kawasaki Disease Clinical Research Group, with validation performed at Chiba University and Tokyo Women's Medical University. A predictive model was developed using data from a primary cohort (*n* = 970) and validated in both internal (*n* = 333) and external (*n* = 262) cohorts. Multivariate analysis identified three predictors of post-treatment CAA presence: maximum pre-treatment *Z*-score ≥1.6 (2 points), albumin level ≤3.1 g/dL (1 point), and age ≤12 months (1 point). A total score of ≥2 predicted CAA with 84.2% sensitivity and 60.8% specificity in the development cohort, with similar performance validated in the internal and external cohorts (area under the receiver operating characteristic curve: both 0.88).

**Conclusions:**

The developed model accurately predicts post-treatment CAA presence, emphasizing the importance of early coronary *Z*-score assessment. It could guide intensive initial therapies to reduce CAA incidence, supporting KD management. However, further validation in diverse populations is recommended.

## Introduction

Kawasaki disease (KD), first described by Tomisaku Kawasaki in 1967 (1), is an acute systemic vasculitis primarily affecting the coronary arteries. Although KD is common in East Asia, cases have been reported worldwide. The most serious complications are coronary artery abnormality (CAA), including coronary arterial dilatation, aneurysm, and stenosis. Notably, approximately 25% of patients with KD who remain untreated develop CAA, increasing the risk of thrombus formation and myocardial infarction (2). Consequently, the sequelae of KD are leading causes of acquired heart disease in children and adolescents in developed countries.

Intravenous immunoglobulin (IVIG) treatment during the acute phase of KD markedly reduces CAA incidence. However, a national survey in Japan revealed that approximately 2.5% of Japanese patients with KD still develop CAA (3), with no declining trend observed over the past decade (4). Current Japanese guidelines, issued by the Japanese Society of Pediatric Cardiology and Cardiac Surgery (3), recommend intensified initial therapy with IVIG plus steroids or cyclosporine for patients predicted to be IVIG resistant based on an IVIG resistance prediction score (5–7). However, some patients with KD develop CAA despite responding well to initial IVIG (8, 9), whereas others remain CAA-free while showing no response to IVIG. Thus, IVIG resistance alone is not a highly accurate predictor of CAA development.

Coronary *Z*-scores, adjusted for sex and body size, have provided a more direct and sensitive assessment of coronary artery diameter changes, improving CAA classification compared with absolute luminal dimensions and Japanese Ministry of Health cutoff points.

Several studies have indicated that coronary artery dilatation (*Z*-score ≥2.0 or 2.5) before initial treatment is a risk factor for CAA development (10–14). However, our prior retrospective single-center study in Japan revealed that a coronary *Z*-score threshold for predicting CAA was closer to 1.5, below the commonly used cutoff of 2 (15).

Therefore, identifying direct CAA risk factors, such as coronary *Z*-scores and other clinical parameters, immediately before primary KD treatment initiation is crucial, rather than focusing solely on IVIG resistance. The objective of the present study was to develop and validate a prediction model for CAA using a multicenter retrospective cohort, incorporating both clinical and laboratory variables prior to treatment initiation.

## Methods

### Development cohort

We conducted a retrospective study of electronic medical records from patients with KD diagnosed between April 2009 and March 2020 at eight medical institutions affiliated with the Wakayama Prefecture Kawasaki Disease Clinical Research Group (Wakayama Medical University Hospital, Wakayama Rosai Hospital, Naga Public Hospital, Hashimoto Municipal Hospital, Hidaka Hospital, Kinan Hospital, Shingu Municipal Medical Center, and Hannan Municipal Hospital). These data (i.e., the development cohort) were used to create a prediction model for CAA presence after treatment. KD was diagnosed according to the clinical criteria from the fifth and sixth diagnostic guidelines established by the Japan Kawasaki Disease Research Committee (16, 17). The first day of illness was defined as the first day of fever (≥37.5°C).

Patients were excluded from the study if they (1) did not receive IVIG treatment or did not follow our treatment protocol (detailed below) (2), had recurrent KD (3), lacked pre-treatment blood test or follow-up echocardiographic data (4), had incomplete electronic medical records, or (5) did not have recorded height measurements.

All enrolled patients received the following treatment protocol: IVIG (2 g/kg/day for 24 h) + aspirin (30 mg/kg/day) as the first-line therapy; IVIG-resistant patients [persistent or recrudescent fever (axillary temperature ≥37.5°C) 24 h after the first IVIG administration] received a second IVIG dose (2 g/kg/day for 24 h) as a second-line therapy; and patients resistant to the second IVIG administration [persistent or recrudescent fever (axillary temperature ≥37.5°C) after IVIG completion] were treated with oral cyclosporine A (5 mg/kg/day) as a third-line therapy.

This study was approved by the Ethics Committee of the Wakayama Medical University (approval no. 2801) and by the Institutional Review Board or Independent Ethics Committee of each participating medical institution.

### Internal and external validation cohorts

Two validation cohorts were enrolled to assess the external and internal validity of the CAA prediction model. The internal validation cohort (VC1) comprised electronic medical records from patients with KD diagnosed at eight participating medical institutions between April 2020 and September 2023. The external validation cohort (VC2) included patients diagnosed at the Department of Pediatrics of Chiba University and Tokyo Women's Medical University Yachiyo Medical Center between January 2017 and December 2019. The same exclusion criteria applied to the development cohort were used for the validation cohorts.

### Data collection

Medical records included patient demographics, clinical characteristics, medications, sequential echocardiographic findings, and clinical laboratory test results prior to the initial treatment. Laboratory tests included white blood cell count, neutrophil percentage, hematocrit, platelet count, aspartate aminotransferase, alanine aminotransferase, total bilirubin, serum albumin, serum sodium, and C-reactive protein (CRP). Variable selection was based on previous KD scoring systems (5–7, 18).

Echocardiographic data were collected for the right coronary artery, left anterior descending artery, and left circumflex artery in two phases: pre-treatment (at KD diagnosis or before IVIG treatment) and convalescent (post-treatment; approximately 4 weeks after KD onset). Left main coronary artery was excluded from the analysis due to frequent anatomical variations (2). *Z*-scores for these coronary dimensions were retrospectively calculated using the *Z*-score calculator (version 4.0 Full, LMS_Z_Score) (19). The highest *Z*-score among the three arteries in each phase was recorded as Zmax. CAA was defined as the post-treatment presence of CAA, based on a Zmax ≥2.5. Consequently, this definition could include patients who already had CAA before treatment (pre-treatment Zmax ≥2.5). The model was developed to predict the post-treatment presence of CAA, encompassing both new-onset and persistent cases.

### Statistical analysis

Continuous variables are reported as medians (interquartile ranges), and categorical variables are presented as frequencies and percentages. Statistical analyses were performed using JMP Pro version 14 (SAS Institute Japan Ltd., Tokyo, Japan). Fisher's exact test was used to analyze categorical variables, whereas the Mann–Whitney *U* test was employed to analyze continuous variables. For multiple comparisons, a Kruskal–Wallis test was initially performed to detect overall significance between groups. When significant, Dunn's test with Bonferroni correction was applied for pairwise comparisons.

Variables with *P* < 0.20 in univariate analysis were considered for multivariable modeling. Multivariable logistic regression analysis was applied to assess independent predictors of CAA, with bilateral *P* values < 0.05 indicating statistical significance. Odds ratios and 95% confidence intervals were calculated. Multicollinearity was assessed based on the variance inflation factor (VIF) of each variable using SPSS version 25 (SPSS Inc., Chicago, IL, USA).

Receiver operating characteristic (ROC) curves were used to determine cutoff values for significant independent predictors of CAA presence after treatment. Predictive variables were assigned scores (1 or 2) based on regression coefficients (RCs), forming a CAA prediction score using pre-treatment clinical data. Sensitivity and specificity were assessed via ROC curves. Model discrimination was evaluated using the area under the ROC curve (AUC), and model performance was additionally assessed using confusion matrices. Furthermore, a sensitivity analysis was conducted using the same method, excluding cases with pre-treatment CAA from the development cohort. The Hosmer–Lemeshow test was used to assess regression model goodness-of-fit using SPSS version 25 (SPSS Inc., Chicago, IL), with *P* > 0.05 indicating no significant deviation between the model and observed event rates.

## Results

### Patient characteristics

In total, 1,230 patients diagnosed with KD at eight medical institutions during the study period were included in the development cohort. The validation cohorts comprised 321 and 413 patients with KD in VC1 and VC2, respectively.

**Figure 1:**
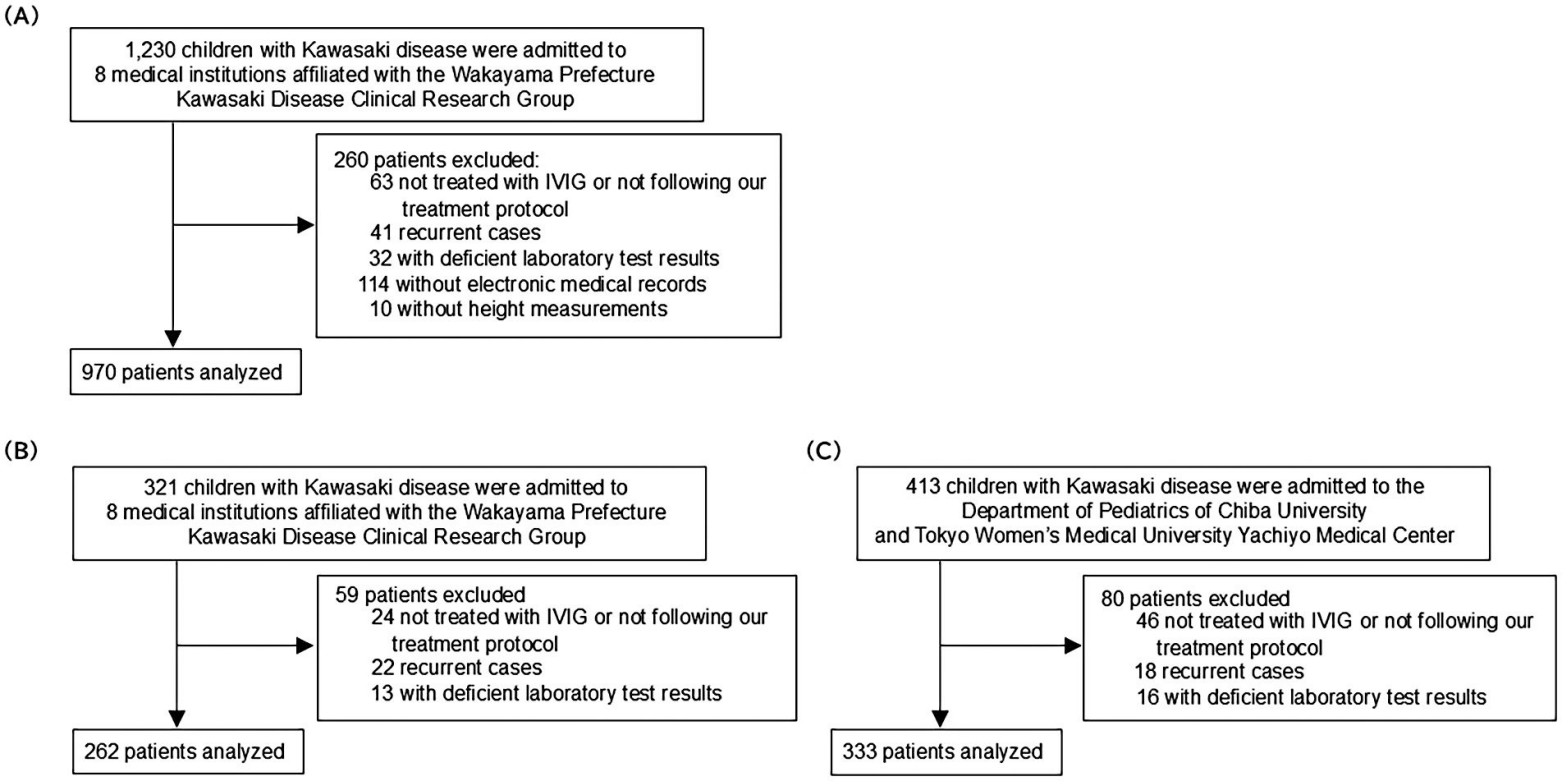
Study flow chart. **(A)** Study flow chart for the development cohort. **(B)** Study flow chart for the internal validation cohort. **(C)** Study flow chart for the external validation cohort. IVIG, intravenous immunoglobulin.

Exclusion criteria led to the removal of patients who did not follow the treatment protocol (*n* = 63), had recurrent KD (*n* = 41), lacked echocardiographic follow-up data (*n* = 32), had incomplete medical records (*n* = 114), or lacked height measurement records (*n* = 10). Consequently, 970, 262, and 333 patients in the development cohort, VC1, and VC2 were analyzed, respectively (Figures 1A–C).

### Predictive model for CAA presence after treatment

In the development cohort, demographic, clinical, treatment, and echocardiographic data at treatment initiation were stratified by CAA status (Table 1). Univariate analysis identified younger age at fever onset, lower hematocrit, platelet count, and albumin levels, and higher CRP and pre-Zmax as significant predictors of CAA (*P* < 0.20). The VIF values for these variables were all <2.0.

**Table 1:** Summary of clinical data in patients with Kawasaki disease stratified by the presence of coronary artery abnormality (CAA).

Variables	Total (*n* = 970)	No CAA (*n* = 913)	CAA (*n* = 57)	*P* value
	Mean (IQR) or No. (%)			
Age, months	25 [13–44]	26 [14–44]	13 [7–28]	<.0001
Male sex, No. (%)	559 (57.6)	524 (57.3)	35 (61.4)	.58
Days from fever onset to 1st IVIG, days	5 [4–5]	5 [4–5]	5 [4–6]	.83
Complete KD, No. (%)	861 (88.7)	809 (88.6)	52 (91.2)	.66
1st IVIG resistance, No. (%)	251 (25.8)	218 (23.8)	33 (57.8)	<.0001
Laboratory data				
WBC,/μL	13,500 [10,677–16,612]	13,515 [10,700–16,612]	12,700 [9,655–16,650]	.39
Neutrophil, %	68.5 [56.1–78.6]	68.5 [56.0–78.5]	68.5 [58.2–79.5]	.42
Hct, %	33.8 [31.8–35.7]	33.8 [31.9–35.7]	32.4 [30.1–34.5]	..0001
Plt, × 10^4^/μL	32.2 [26.6–38.6]	32.4 [26.8–38.6]	28.0 [25.8–36.2]	.047
Alb, g/dL	3.6 [3.3–3.9]	3.6 [3.3–3.9]	3.3 [2.9–3.6]	<.0001
AST, U/L	35 [26–76]	35 [26–76]	36 [26–79]	.84
ALT, U/L	22 [13–88]	21 [13–88]	31 [16–91.5]	.08
T-bil, mg/dL	0.6 [0.5–0.9]	0.6 [0.5–0.9]	0.7 [0.4–1.1]	.21
Na, mEq/L	134.1 [132.2–136.0]	134.1 [132.2–136.0]	134.0 [131.2–136.0]	.31
CRP, mg/dL	6.5 [3.94–10.0]	6.4 [3.8–9.9]	8.7 [5.7–11.0]	.004
Echocardiography data				
Pre-Zmax	1.36 [0.82–2.04]	1.31 [0.79–1.97]	2.26 [1.69–3.56]	<.0001
Pre-Zmax ≥2.5, No. (%)	130 (13.4)	106 (11.6)	24 (42.1)	<.0001

IQR, interquartile range; KD, Kawasaki disease; IVIG, intravenous immunoglobulin; WBC, white blood cell count; Hct, hematocrit; Plt, platelet count; Alb, albumin; AST, aspartate aminotransferase; ALT, alanine aminotransferase; T-bil, total bilirubin; Na, sodium; CRP, C-reactive protein; Pre-Zmax, maximum coronary artery *Z*-score at KD diagnosis or before IVIG treatment.

The median time from fever onset to treatment initiation was 5 days, with no significant difference between the CAA and no-CAA groups. In the CAA group, 33 patients (57.8%) showed refractoriness to initial IVIG therapy, and 24 patients (42.1%) had CAA before initial treatment.

Multivariate analysis confirmed that younger age at diagnosis, lower baseline albumin level, and higher pre-Zmax were independent CAA predictors (Table 2A). ROC curve analysis determined the following cutoff values: pre-Zmax ≥1.6, albumin level ≤3.1 g/dL, and age ≤12 months (Supplementary Figure 1). Each cutoff value was significantly associated with CAA: pre-Zmax ≥1.6 (RC: 0.868; *P* < 0.0001), albumin level ≤3.1 g/dL (RC: 0.699; *P* < 0.0001), and age ≤12 months (RC: 0.489; *P* = 0.0008) (Table 2B).

**Table 2:** Multivariate logistic regression analysis of independent predictors of coronary artery abnormality (CAA) with cutoff values for significant predictors.

(A) Multivariate logistic regression analysis of independent CAA predictors		
Variables	Multivariate analysis	
	Adjusted OR (95% CI)	*P* value
Age, months	0.97 (0.96–0.99)	.005
Hct, %	0.93 (0.83–1.05)	.29
Plt, × 10^4^/μL	0.97 (0.94–1.00)	.051
Alb, g/dL	0.23 (0.12–0.46)	<.0001
ALT, U/L	1.00 (0.99–1.00)	.61
CRP, mg/dL	1.00 (0.94–1.07)	.75
Pre-Zmax	2.57 (1.90–3.48)	<.0001

(B) Cutoff values, regression coefficients, and odds ratios of significant CAA predictors				
Data	Value	Regression coefficient	OR (95% CI)	*P* value
Pre-Zmax	≥1.6	0.868	5.67 (2.96–11.8)	<.0001
Albumin (g/dL)	≤3.1	0.699	4.04 (2.22–7.27)	<.0001
Age in months	≤12	0.489	2.66 (1.49–4.72)	.0008

CI, confidence interval; Hct, hematocrit; Plt, platelet count; Alb, albumin; ALT, alanine aminotransferase; CRP, C-reactive protein; Pre-Zmax, maximum coronary artery *Z*-score at KD diagnosis or before IVIG treatment; OR, odds ratio.

**Figure 2:**
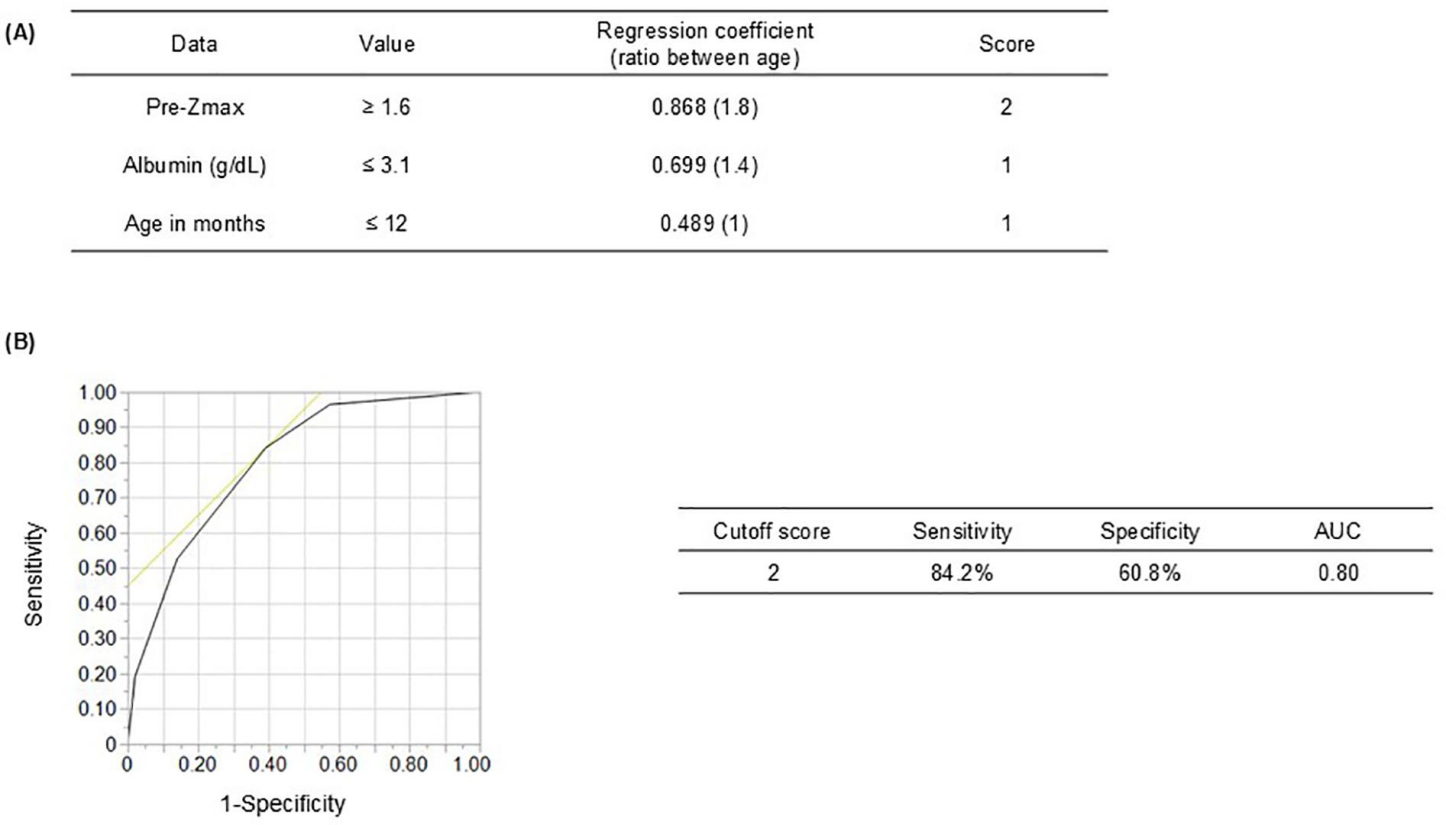
Receiver operating characteristic curve showing the cutoff scores for coronary artery abnormality (CAA) predictors. **(A)** Prediction scores for CAA development due to Kawasaki disease considering three clinical variables: Pre-Zmax (≥1.6, 2 points), serum albumin level (<3.1 g/dL, 1 point), and age in months (≤12, 1 point). **(B)** At a cutoff score of ≥2, sensitivity and specificity were 84.2 and 60.8%, respectively. Pre-Zmax, pre-treatment (at KD diagnosis or before IVIG treatment) maximum coronary artery *Z*-score; AUC, area under the receiver operating characteristic curve.

Scores for CAA prediction were assigned based on the ratios of RC values: pre-Zmax ≥1.6 scored 2, whereas albumin level ≤3.1 g/dL and age ≤12 months each scored 1 (Figure 2A). The total score cutoff for predicting CAA, determined using ROC curve analysis, was ≥2, yielding a sensitivity and specificity of 84.2% and 60.8%, respectively (AUC: 0.80) (Figure 2B). The positive predictive value (PPV) was 11.8%, and the negative predictive value was 98.4% (Supplementary Figure 2A). The Hosmer–Lemeshow statistic (*P* = 0.73) indicated no significant deviation from model fit. Furthermore, when a sensitivity analysis was performed excluding cases with CAA before initial treatment (pre-treatment Zmax ≥2.5), the sensitivity and specificity were 72.9% and 64.8%, respectively (AUC: 0.74) (Supplementary Figure 2B). The RCs for albumin and age increased, whereas that for pre-Zmax decreased (Supplementary Figure 2C). These results suggest that the contribution of pre-treatment coronary *Z*-score may be relatively llimitited in patients without pre-treatment CAA, although the model appears to retain its overall predictive power.

### Internal and external validation of the CAA prediction model

**Figure 3:**
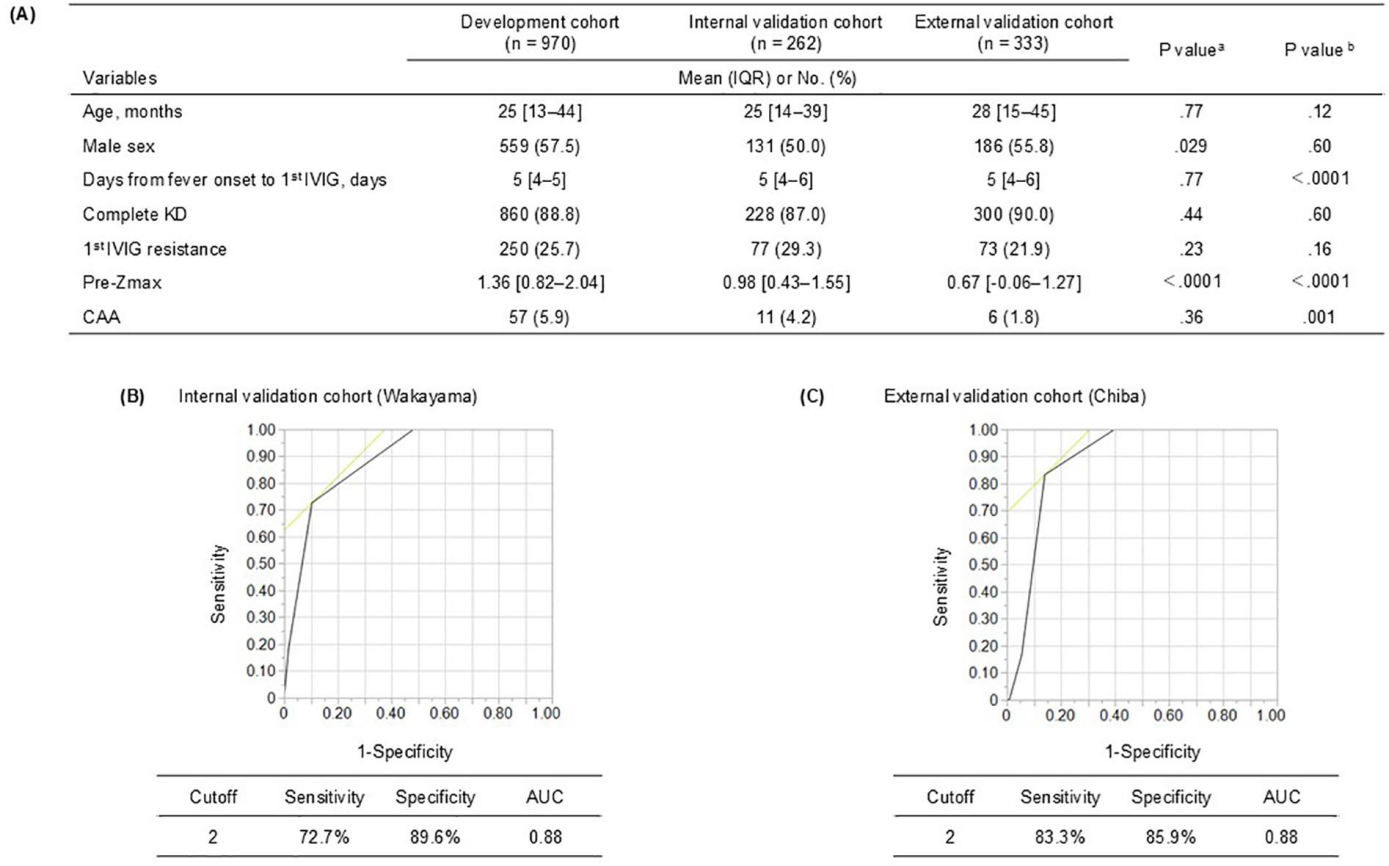
Adaptation of the coronary artery abnormality (CAA) prediction score model in the validation cohorts. **(A)** Baseline characteristics and echocardiography studies in the development and validation cohorts. **(B)** In the internal validation cohort, a cutoff score of ≥2, had a sensitivity and specificity of 72.7% and 89.6%, respectively. **(C)** In the external validation cohort, a cutoff score of ≥2, had a sensitivity and specificity of 83.3% and 85.9%, respectively. KD, Kawasaki disease; IVIG, intravenous immunoglobulin; Pre-Zmax, pre-treatment (at KD diagnosis or before IVIG treatment) maximum coronary artery *Z*-score; AUC, area under the receiver operating characteristic curve. ^a^Outcomes in the development vs. internal validation cohort. ^b^Outcomes in the development vs. external validation cohort.

Clinical characteristics that differed significantly between the development cohort and either validation cohort included the maximum pre-treatment *Z*-score (pre-Zmax). The incidence of CAA was 5.9% (*n* = 57) in the development cohort, 4.2% (*n* = 11) in VC1, and 1.8% (*n* = 6) in VC2, with VC2 showing a significantly lower incidence compared to the development cohort (Figure 3A).

The model's internal validity was evaluated in VC1, despite significant differences in sex and pre-Zmax between the development cohort and VC1. The model demonstrated excellent discrimination (AUC: 0.88), 72.7% sensitivity, and 89.6% specificity (Figure 3B). The Hosmer–Lemeshow statistic was nonsignificant (*P* = 0.83), indicating good fit.

External validity was evaluated in VC2, where the model achieved strong discrimination despite the cohort's low CAA incidence. AUC was 0.88, with 83.3% sensitivity and 85.9% specificity (Figure 3C). The Hosmer–Lemeshow statistic (*P* = 0.25) showed no significant deviation.

Among 1,565 KD cases across three cohorts, 74 remained CAA. Of these, 61 cases (82.4%) were classified as high-risk by our CAA prediction model (Supplementary Figure 2).

## Discussion

This study demonstrates the potential for predicting CAA using clinical parameters obtained before the initiation of initial treatment for KD. The findings suggest that the developed predictive model could help identify high-risk cases and facilitate more targeted initial treatment strategies to prevent CAA.

In Japan, patients resistant to IVIG therapy have historically been considered at high-risk for developing CAA. Accordingly, Japanese guidelines recommend intensified initial therapy for KD cases with a high IVIG resistance risk score (3). Although a nationwide KD survey reported a decline in CAA incidence to approximately 2.5%, this rate has plateaued over the past decade (4).

Although IVIG resistance has been considered a risk factor for CAA, only 57.8% of patients in our development cohort who remained CAA were IVIG resistant. This proportion is similar to that reported in other large-scale retrospective Japanese studies (51.0%) (20). Prior research has also failed to establish a consistent association between IVIG resistance and CAA development (21).

Our analysis revealed that, except for age, the predictors for CAA and IVIG resistance differ (Supplementary Table 1), suggesting that IVIG resistance prediction scores may not directly predict CAA development. This finding raises concerns about the appropriateness of relying on these scores (5–7) to guide intensified initial therapy.

Our findings support the utilization of pre-treatment coronary artery *Z*-scores are, which have been widely recognized as key predictors of CAA, independent of IVIG resistance (10–14). In fact, Matsuoka et al. identified a pre-treatment coronary artery *Z*-score of >2.5 as the sole risk factor for CAA in 325 IVIG-responsive KD cases; however, the score showed limited sensitivity (50%), possibly due to the fact that 31% of their CAA cases had *Z*-scores of <2.0 at diagnosis (9). Therefore, the use of a lower cutoff may be more appropriate in Japanese clinical settings. This discrepancy may stem from the earlier diagnosis of KD in Japan relative to other countries or from differences in *Z*-score calculation methods.

Japanese IVIG resistance prediction scores have shown poor reproducibility outside of Japan (22–25). Conversely, the key variables in our CAA prediction model (i.e., pre-treatment coronary artery *Z*-scores, younger age, and low albumin levels) have been consistently associated with CAA across diverse populations worldwide, suggesting minimal regional or racial variability. A key strength of our prediction model is its high sensitivity in identifying patients at risk of post-treatment presence of CAA. Among the 1,565 patients with KD across 3 cohorts, 74 had CAA. Among these patients, 61 (82.4%) were classified as high-risk based on our CAA prediction model, whereas only 28 (37.8%) were identified as high-risk based on the IVIG resistance prediction (Gunma) score (Supplementary Figure 3).

We have previously reported that infants aged under 12 months have a lower incidence of IVIG resistance compared with other age groups but a higher risk of developing CAA (26). Although the underlying mechanisms remain unclear, they may involve factors unique to infancy, such as the structural fragility of coronary artery walls, inflammatory processes localized to the coronary arteries, and systemic febrile inflammatory responses dissociated from immune responses due to developmental vulnerabilities. Therefore, particularly in younger age groups, treatment decisions should be based on direct CAA predictors rather than IVIG treatment response.

Albumin levels are not included in common IVIG resistance prediction scores used in Japan (e.g., Gunma, Osaka, and Kurume scores). These levels likely reflect the severity of early-stage vasculitis rather than IVIG resistance. Increased vascular permeability may worsen vascular wall destruction by exacerbating edema, creating a vicious cycle.

Although our definition of CAA encompasses both new-onset and persistent cases, it reflects the real-world clinical challenge of identifying patients at risk of having significant coronary abnormalities after treatment. In KD, all patients receive prompt, standardized treatment upon diagnosis, including IVIG and, in selected cases, intensive immunosuppressive therapy. Therefore, the natural progression to CAA may be interrupted, complicating model evaluation. Some patients classified as false negatives may, in fact, have benefited from early treatment that prevented the onset of CAA. This inherent limitation may partly explain the observed low PPV of our model.

Prior to treatment initiation, it is critical to identify patients with KD at high-risk for CAA based on specific predictors, including *Z*-scores, age, and albumin levels, rather than relying on IVIG resistance. Our findings underscore the clinical utility of a predictive model incorporating these easily measurable and frequently assessed factors in KD management. Future studies are warranted to validate whether targeted interventions, guided by model-predicted high-risk status, can improve patient outcomes.

### Study limitations

In Japan, KD diagnosis and treatment involve not only pediatric cardiologists but also general pediatricians, which could affect the reproducibility of coronary artery *Z*-scores. There was no standardization of measurement methods between facilities or examiners, and the varying echocardiographic equipment used in this study could also impact consistency. Additionally, in the development cohort, patients were diagnosed according to either the 5th and 6th editions of the Japanese KD guidelines. It is possible that differences in diagnostic criteria may have influenced the date of IVIG administration. However, validation in two independent cohorts indicates that the CAA presence prediction score, including pre-Zmax, remains applicable even when general pediatricians perform coronary artery measurements. Notably, the findings in this study are specific to the treatment protocol used, and coronary *Z*-scores were calculated using the Coronary *Z*-Score Calculator (Version 4.0 LMS_Z_Score). All variables in this predictive model were not standardized using *Z*-score for clinical interpretability. This lack of standardization may limit direct comparisons between RCs. The development cohort for this model included patients with CAA before treatment. The strong correlation between pre-treatment and post-treatment *Z*-scores may have led to an overestimation of model performance. To evaluate the robustness of the model, we therefore conducted a sensitivity analysis excluding patients with pre-treatment CAA. As expected given the change in cohort structure, the RCs shifted in the sensitivity analysis. However, this does not undermine the overall utility of the model. Nonetheless, this limitation should be acknowledged in interpreting the results. Finally, whether intensive therapy is beneficial for patients predicted to develop CAA remains an open question requiring further investigation in future studies.

## Conclusions

We developed and validated a predictive model for CAA presence after treatment in two cohorts of Japanese patients with KD prior to treatment initiation. Our findings indicate that age, albumin level, and pre-treatment coronary *Z*-score are independently associated with CAA presence after treatment. This simple scoring model could be a valuable tool for guiding intensified initial therapy to reduce the post-treatment incidence of CAA. Future studies should validate this CAA presence prediction score in larger and more diverse medical settings in Japan to assess its accuracy and generalizability.

## Data Availability

The original contributions presented in the study are included in the article/Supplementary Material, further inquiries can be directed to the corresponding author.
