# Supplementary material for: A prediction model for post-treatment presence of coronary artery abnormality before initial treatment in Kawasaki disease in Japan
Source: Front Pediatr. 2025 Dec 2;13:1647195. doi: 10.3389/fped.2025.1647195 (PMC12705635; doi:10.3389/fped.2025.1647195)
Supplement: Supplementary file 1 [file Supplementaryfile1.pdf]

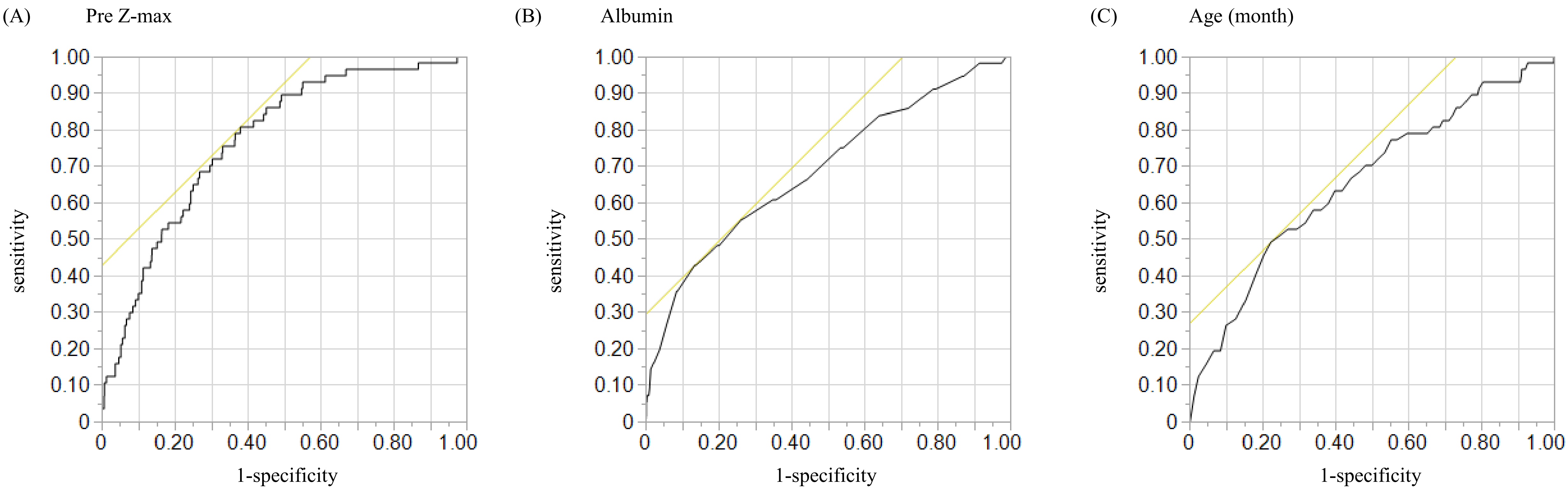

Cutoff	sensitivity	specificity	AUC
1.6	80.7%	57.3%	0.76

Cutoff	sensitivity	specificity	AUC
3.1	42.8%	86.4%	0.68

Cutoff	sensitivity	specificity	AUC
12.0	49.1%	77.6%	0.65

Supplementary Figure 1. Receiver operating characteristic curves for the cutoff values of significant independent coronary artery abnormality predictors. For Pre-Zmax, using a cutoff of ≥1.6, sensitivity and specificity were 80.7% and 57.3%, respectively (A). For albumin, using a cutoff of ≤3.1, sensitivity and specificity were 42.8% and 86.4%, respectively (B). For months of age, using a cutoff of ≤12, sensitivity and specificity were 49.1% and 77.6%, respectively (C). Pre-Zmax, pretreatment (at KD diagnosis or before IVIG treatment) maximum coronary artery Zscore; AUC, area under the receiver operating characteristic curve.

(A)

	No CAA	CAA
Score ≥2 (Predicted CAA)	358 (False Positive)	48 (True Positive)
Score <2 (Predicted no CAA)	555 (True Negative)	9 (False Negative)

(B)

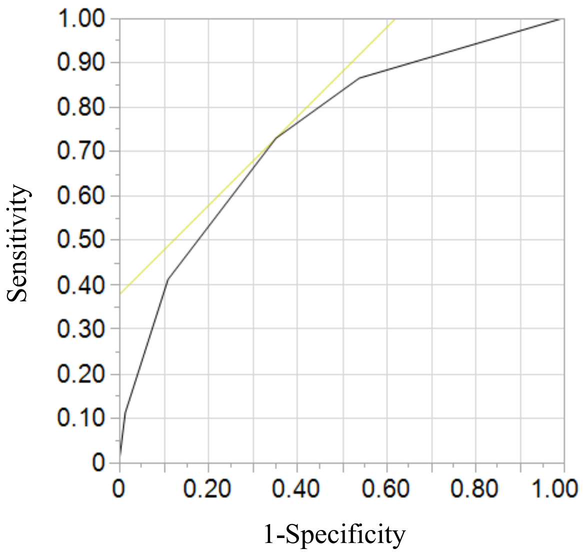

Cutoff score	Sensitivity	Specificity	AUC
2	72.9%	64.8%	0.74

(C)

Data	Value	Regression coefficient (ratio between age)
Pre-Zmax	≥ 1.6	0.697 (1.3)
Albumin (g/dL)	≤ 3.1	0.768 (1.4)
Age in months	≤ 12	0.554 (1)

Supplementary Figure 2. (A) Confusion matrix for the development cohort. (B) Receiver operating characteristic curve showing the cutoff score of the prediction model for the presence of post-treatment coronary artery abnormalities (CAA), excluding cases with pre-treatment CAA. (C) Regression coefficients of the prediction model for the presence of post-treatment CAA, excluding cases with pre-treatment CAA.

Abbreviations: Pre-Zmax, maximum coronary artery Z-score at KD diagnosis or before IVIG treatment; AUC, area under the receiver operating characteristic curve.

Supplementary Table 1. Multivariate analysis of risk factors for initial IVIG resistance in the development cohort.

	Multivariate	
	Adjusted OR (95% CI)	P value
Male sex	1.53 (1.10-2.14)	.01
Neutrophil, (%)	1.07 (1.05-1.08)	<.001
Plt , ($\times 10^4$/μL)	0.96 (0.94-0.98)	<.001
T-bil, (mg/dL)	1.64 (1.33-2.03)	<.001
Age, (months)	0.97 (0.96-0.97)	<.001

CI, confidence interval; CAA, coronary artery abnormality; IVIG, intravenous immunoglobulin; Plt, platelet count; Alb, albumin; T-bil, total bilirubin.

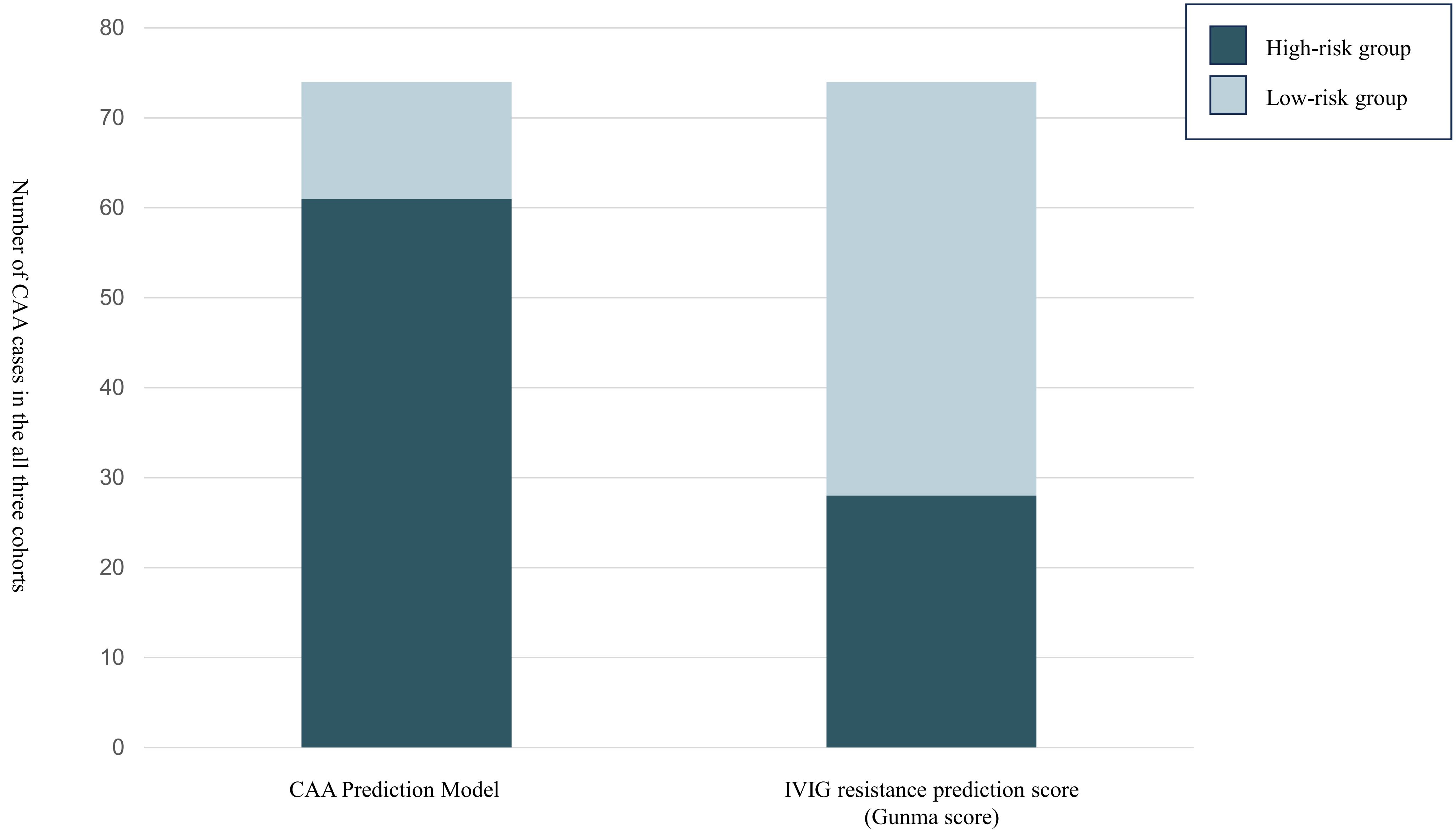

Supplementary Figure 3. Number of high- and low-risk coronary artery abnormality (CAA) cases in the prediction model and with IVIG resistance prediction scores.
IVIG, intravenous immunoglobulin
